# Machine Learning-Aided Analysis of the Rolling and Recrystallization Textures of Pure Iron with Different Cold Reduction Ratios and Cold-Rolling Directions

**DOI:** 10.3390/ma17143402

**Published:** 2024-07-10

**Authors:** Takumi Sumida, Keiya Sugiura, Toshio Ogawa, Ta-Te Chen, Fei Sun, Yoshitaka Adachi, Atsushi Yamaguchi, Yukihiro Matsubara

**Affiliations:** 1 Department of Materials Design Innovation Engineering, Graduate School of Engineering, Nagoya University, Furo-cho, Chikusa-ku, Nagoya 464-8603, Japan; 2 Department of Mechanical Engineering, Faculty of Engineering, Aichi Institute of Technology, 1247 Yachigusa, Yakusa-cho, Toyota 470-0392, Japan; 3 Asahi-Seiki Manufacturing Co., Ltd., 5050-1 Shindenbora, Asahimae-cho, Owariasahi 488-8655, Japan

**Keywords:** texture, pure iron, cold-rolling, machine learning

## Abstract

We performed a machine learning-aided analysis of the rolling and recrystallization textures in pure iron with different cold reduction ratios and cold-rolling directions. Five types of specimens with different cold reduction ratios and cold-rolling directions were prepared. The effect of two-way cold-rolling on the rolling texture was small at cold reduction ratios different from 60%. The cold reduction ratio in each stage hardly affected the texture evolution during cold-rolling and subsequent short-term annealing. In the case of long-term annealing, although abnormal grain growth occurred, the crystal orientation of the grains varied. Moreover, the direction of cold-rolling in each stage also hardly affected the texture evolution during cold-rolling and subsequent short-term annealing. During long-term annealing, sheets with the same cold-rolling direction in the as-received state and in the first stage showed the texture evolution of conventional one-way cold-rolled pure iron. Additionally, we conducted a machine learning-aided analysis of rolling and recrystallization textures. Using cold-rolling and annealing conditions as the input data and the degree of Goss orientation development as the output data, we constructed high-accuracy regression models using artificial neural networks and XGBoost. We also revealed that the annealing temperature is the dominant factor in the nucleation of Goss grains.

## 1. Introduction

Microstructural control of metals can improve their properties [1,2,3]. Textural control of metals is also crucial for improving their mechanical and magnetic properties [4,5,6]. For instance, the development of {111}//normal direction (ND) fiber texture (γ-fiber) improves the deep drawability of steels [4]. Moreover, the development of <100>//rolling direction (RD) can improve the magnetic properties of steels [5]. Thus, multiple methods for controlling the texture of various steels through optimization of the type of alloying elements and manufacturing processes have already been established [7,8,9,10].

In the case of pure iron, the texture is controlled only by the manufacturing process because pure iron does not contain alloying elements. Therefore, methods for controlling the texture of pure iron are limited compared with those of various other steels. Tomita et al. [11] demonstrated that the cold-rolling of pure iron at a reduction ratio of 99.8% leads to the extreme development of α-fiber. Furthermore, Okai et al. [12] revealed that two-stage cold-rolling and annealing result in a near-cube orientation. Recently, we found that two-way cold-rolling with subsequent short-term annealing results in the randomization of the recrystallization texture and formation of {110} <001> (Goss orientation) grains [13]. Additionally, we revealed that two-way cold-rolling with subsequent long-term annealing leads to abnormal Goss grain growth [14]. However, the texture evolution of pure iron with various cold reduction ratios and cold-rolling directions has not been sufficiently investigated in our previous studies, indicating that there is still potential for texture control in pure iron. Therefore, we can develop unique textures that have not previously been observed in pure iron.

Experiments alone are insufficient to further expand the range of methods for controlling the texture of pure iron. In recent years, material research using machine learning has progressed remarkably [15,16,17,18,19,20,21,22]. For instance, machine learning has been applied to the prediction of fatigue [18,19] and creep life [20], segmentation for microstructure [21], and detection of crack initiation on fracture surfaces [22]. Therefore, machine learning is expected to be useful for expanding the possibilities of textural control of pure iron.

The present study had two main objectives. First, we experimentally investigated the rolling and recrystallization textures in pure iron with different cold reduction ratios and cold-rolling directions. Second, we performed a machine learning-aided analysis of rolling and recrystallization textures in pure iron. In this study, using experiments and machine learning, we elucidated the texture evolution of pure iron after two-way cold-rolling and subsequent annealing.

## 2. Materials and Methods

**Figure 1:**
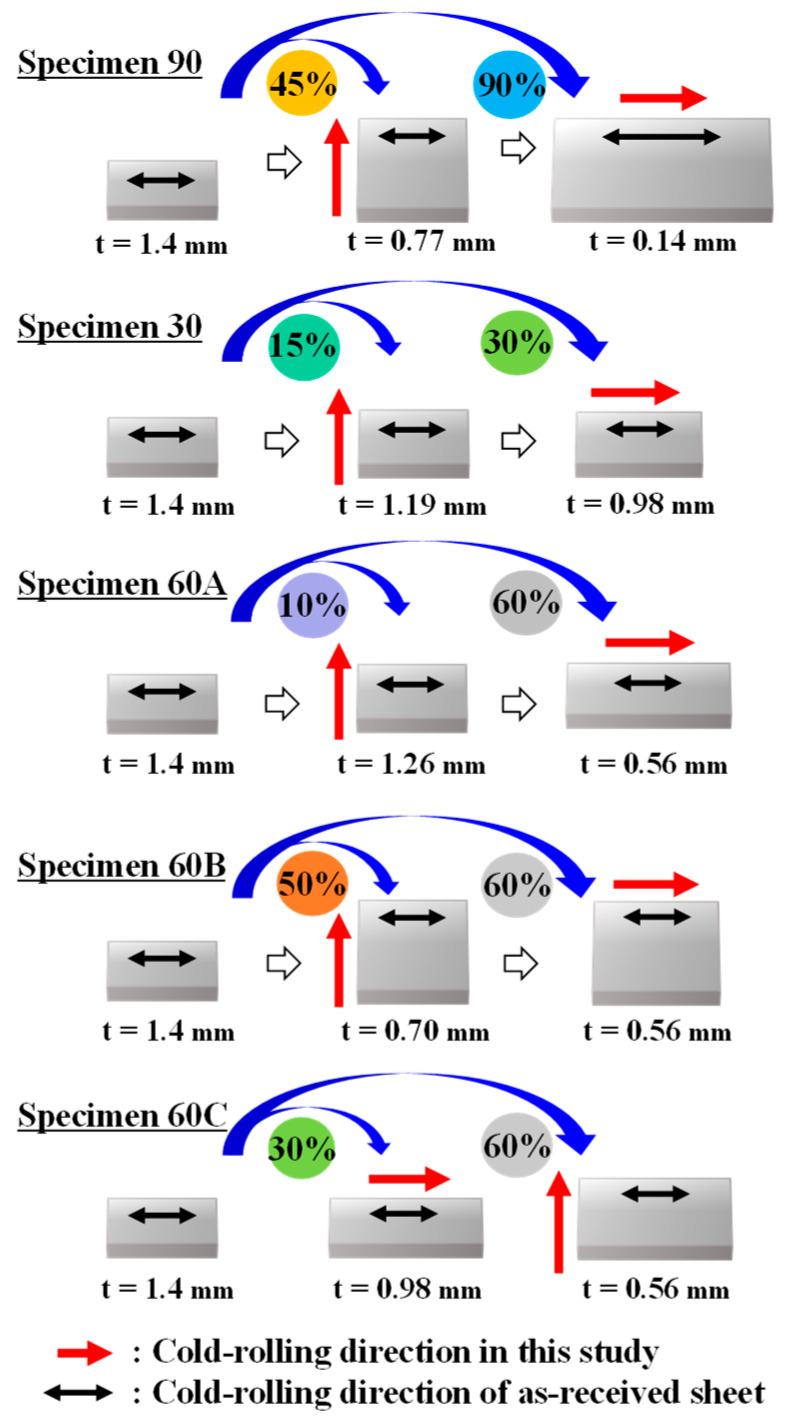
Schematics of the cold-rolling conditions (Cold reduction ratios from the start to the end of the blue arrows are indicated near the end of the arrow).

Pure iron sheets (thickness: 1.4 mm) were used. Details of the as-received pure iron sheets are provided in our previous studies [13,14]. As shown in Figure 1, the sheets were cold-rolled under various conditions to vary the state and amount of strain. Specimens 90 and 30 were cold-rolled to a thickness of 0.77 and 1.19 mm, respectively, in a vertical direction against the cold-rolling direction of the as-received sheet and then to a thickness of 0.14 and 0.98 mm, respectively, in the cold-rolling direction of the as-received sheet. Specimens 60A and 60B were cold-rolled to a thickness of 1.26 and 0.70 mm, respectively, in a vertical direction against the cold-rolling direction of the as-received sheet and then to a thickness of 0.56 mm in the cold-rolling direction of the as-received sheet. Specimen 60C was cold-rolled to a thickness of 0.98 mm in the cold-rolling direction of the as-received sheet and then to a thickness of 0.56 mm in a vertical direction against the cold-rolling direction of the as-received sheet. In this way, five types of cold-rolled sheets were prepared.

The cold-rolled sheets were cut into 0.14 to 0.98 (thickness) × 10 × 10 mm specimens for annealing. In the short-term annealing treatment, the cold-rolled specimens were heated to 1073 K at a rate of 5 K/s and then water-quenched to room temperature (298 ± 2 K). In the long-term annealing treatment, the cold-rolled specimens were heated to 1123 K at a rate of 0.16 K/s and held for 180 min at the target temperature; subsequently, they were furnace-cooled to 773 K and water-quenched to room temperature. The short-term and long-term annealing was performed to evaluate the recrystallization and grain growth behavior, respectively.

Microstructural and textural analyses in the RD–ND plane were performed on cold-rolled and annealed specimens using electron backscatter diffraction/field emission scanning electron microscopy (EBSD/FESEM) system (JSM-7001FA, JEOL, Tokyo, Japan) and the orientation imaging microscopy (OIM) analysis software (version 7.3.1, TSL solutions, Kanagawa, Japan). The step sizes for the EBSD measurements ranged from 0.5 to 10 μm, and the scan area ranged from 0.10 to 0.70 mm^2^.

Based on the experimental results obtained in the present and previous studies [13,14], we constructed regression models using artificial neural networks (ANNs) [23] and XGBoost [24]. ANNs consist of input, hidden, and output layers, and the hidden layer has several nodes. The number of hidden layers and nodes is crucial for constructing the ANN model. Additionally, XGBoost is one of the most representative ensemble learning algorithms and combines boosting and decision trees. We used both ANN and XGBoost to evaluate the reliability of output data obtained by machine learning. Cold-rolling (cold reduction ratio in each cold-rolling direction and order of cold-rolling direction) and annealing conditions (heating rate, annealing temperature, and holding time at annealing temperature) were used as the input data, and the degree of development of Goss orientation was used as the output data. The orientation distribution function (ODF) intensity of Goss orientation obtained from EBSD results was employed as the degree of development of Goss orientation. In the construction of regression models, 90% of the data (number of data: 72) were used for training, and 10% of the data (number of data: 8) were used for testing. These datasets include data obtained in this study as well as data obtained in our previous studies [13,14]. The ANN model had a single hidden layer and 5 hidden nodes. Additionally, sensitivity analysis [25] and Shapley additive explanations (SHAP) [20,26] were used to quantitatively evaluate the effect of cold-rolling and annealing conditions on the degree of development of Goss orientation. These methods can quantitatively suggest the effect of each input parameter on an output parameter. We used both a sensitivity analysis and SHAP to evaluate the reliability of the obtained results. The connecting weight algorithm was used for the sensitivity analysis, and the details of the algorithm are provided in the literature [27]. Models were constructed and analyzed using Shiny MIPHA (Shinkouseiki Co., Ltd., Fukuoka, Japan) [28]. Shiny MIPHA provides sparse study and regression analysis methods that enable a data-driven properties-to-microstructure-to-processing inverse materials-design approach.

## 3. Results and Discussion

### 3.1. Effect of the Cold Reduction Ratio (Comparison of Specimens 90 and 30)

**Figure 2:**
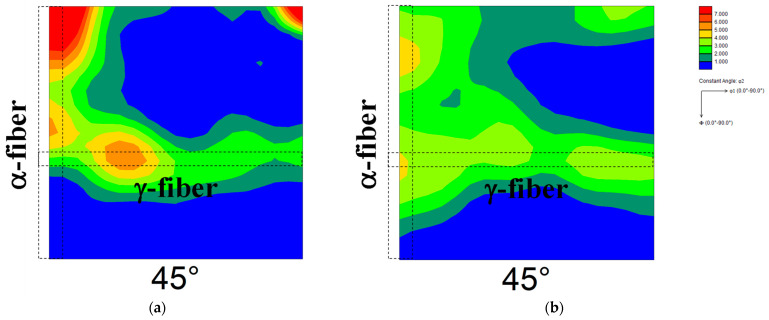
ODF maps of cold-rolled specimens (**a**) 90 and (**b**) 30.

Figure 2 shows the ODF maps of cold-rolled specimens 90 and 30. The highest total cold reduction ratio was found in specimen 90, followed by the specimen used in our previous reports [13,14] and specimen 30 in descending order. In specimen 90 (Figure 2a), the development of γ-fiber and α-fiber was observed, and the degree of α-fiber development was higher than that of γ-fiber development. Both γ-fiber and α-fiber develop in cold-rolled pure iron [29]. Simultaneously, Tomita et al. [11] demonstrated that the cold-rolling of pure iron at a reduction rate above 90% results in the extreme development of α-fiber, which agrees with the result shown in Figure 2a. In contrast, both γ-fiber and α-fiber developed in specimen 30 (Figure 2b), and their degree of development was lower than that in the specimen used in our previous report [13,14]. Zhang et al. [30] reported that the degree of development of γ-fiber and α-fiber in the interstitial free steel decreases with decreasing cold reduction ratio, which agrees with the results obtained in this study.

**Figure 3:**
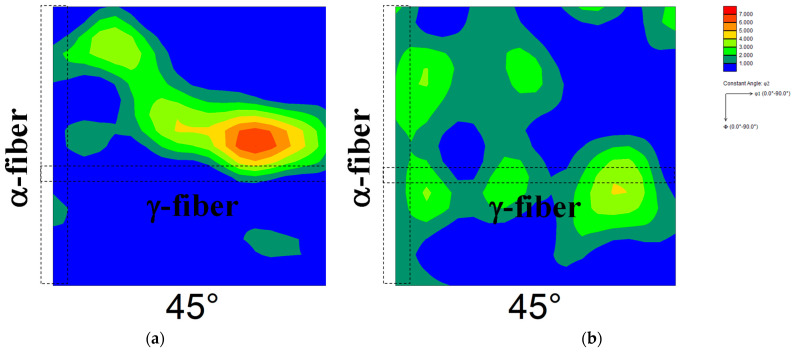
ODF maps of specimens (**a**) 90 and (**b**) 30 annealed at 1073 K.

**Figure 4:**
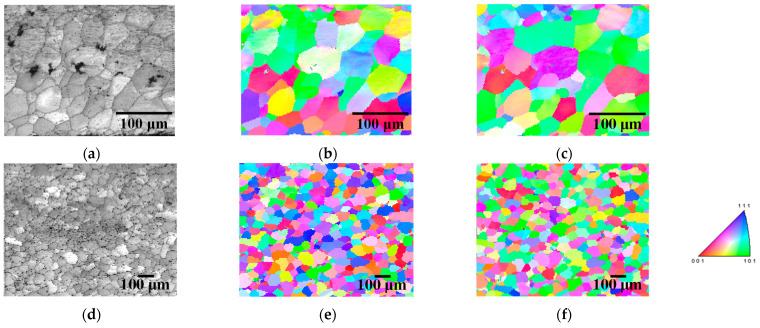
(**a**,**d**) IQ and IPF maps in the (**b**,**e**) normal and (**c**,**f**) rolling directions of specimens 90 and 30 annealed at 1123 K for 180 min.

Figure 3 shows the ODF maps of specimens 90 and 30 annealed at 1073 K. Both γ-fiber and α-fiber slightly developed in both specimens, suggesting that the rolling texture of each specimen was partially retained after short-term annealing. Figure 4 shows the inverse pole figure (IPF) maps of specimens 90 and 30 annealed at 1123 K for 180 min. Abnormal grain growth was not observed in their specimens, even though it was observed in our previous study [14]. Thus, the rolling and recrystallization textures of specimens 90 and 30 are similar to those of conventional one-way cold-rolled pure iron [11,30]. Homma et al. [31] reported the development of Goss orientation in bcc iron at cold reduction ratios between 40% and 60%. According to these results, two-way cold-rolling does not significantly affect the texture evolution at cold reduction ratios different from 60%.

### 3.2. Effect of the Cold Reduction Ratio at Each Stage (Comparison of Specimens 60A and 60B)

**Figure 5:**
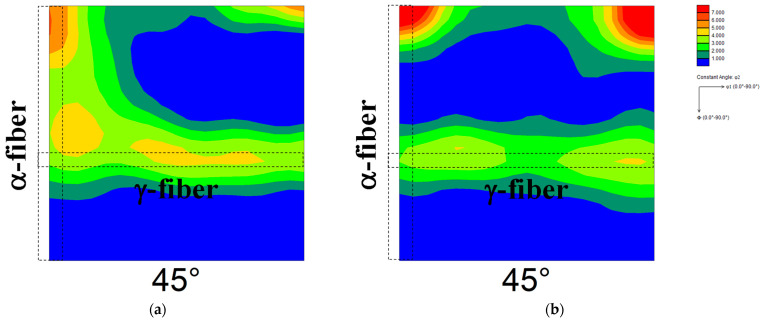
ODF maps of cold-rolled specimens (**a**) 60A and (**b**) 60B.

**Figure 6:**
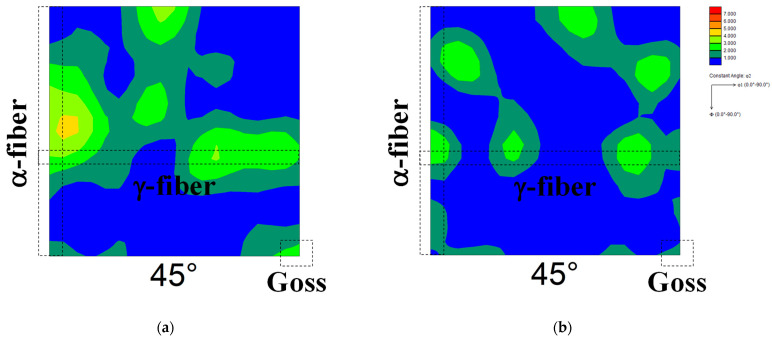
ODF maps of specimens (**a**) 60A and (**b**) 60B annealed at 1073 K.

Figure 5 shows the ODF maps of cold-rolled specimens 60A and 60B. The highest cold reduction ratio in the first stage was found in specimen 60B, followed by the specimen used in our previous report [13,14] and specimen 60A in descending order. The development of γ-fiber and α-fiber was observed irrespective of the cold reduction ratio in the first stage. Figure 6 shows the ODF maps of specimens 60A and 60B annealed at 1073 K. Randomization of recrystallization texture and the emergence of Goss orientation were observed irrespective of the cold reduction ratio in the first stage. We previously confirmed the randomization of recrystallization texture and nucleation of Goss grains in pure iron due to two-way cold-rolling and short-term annealing [13]. Thus, the result shown in Figure 6 is in good agreement with previous findings [13].

**Figure 7:**
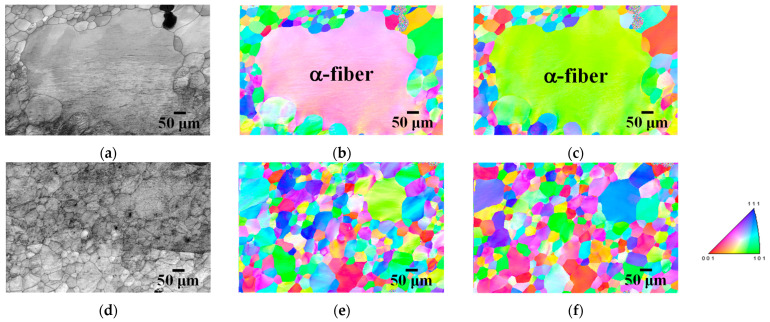
(**a**,**d**) IQ and IPF maps in the (**b**,**e**) normal and (**c**,**f**) rolling directions of specimens 60A and 60B annealed at 1123 K for 180 min.

Figure 7 shows the IPF maps of specimens 60A and 60B annealed at 1123 K for 180 min. Abnormal grain growth was observed irrespective of the cold reduction ratio in the first stage. However, grains with abnormal growth did not necessarily have a Goss orientation. For instance, Figure 7a–c shows the abnormal growth of grains with α-fiber. We previously demonstrated that long-term annealing results in abnormal growth of Goss grains in two-way cold-rolled pure iron [14]. These findings suggest that the cold reduction ratio in each stage hardly affects the texture evolution during cold-rolling and subsequent short-term annealing. However, even though we reported abnormal grain growth during long-term annealing in our previous study [14], the crystal orientation of the grains varies here. Thus, abnormal grain growth during long-term annealing must be further investigated in the future using a greater number of EBSD measurements.

### 3.3. Effect of Cold-Rolling Direction (Specimen 60C)

**Figure 8:**
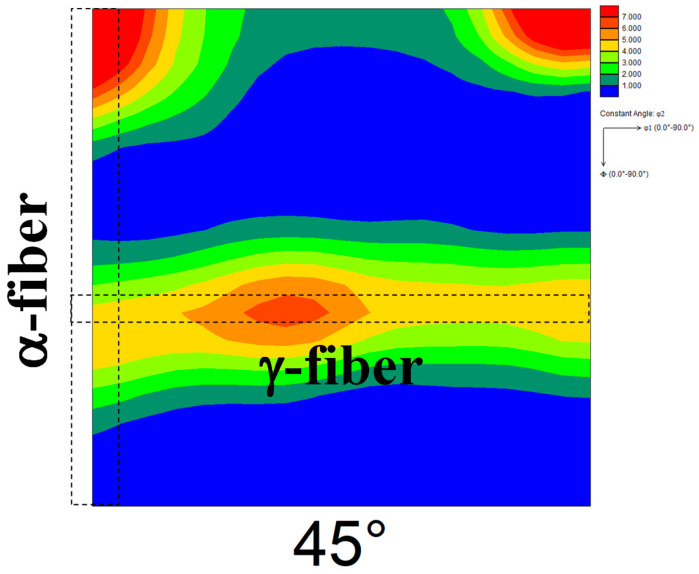
ODF map of the cold-rolled specimen 60C.

**Figure 9:**
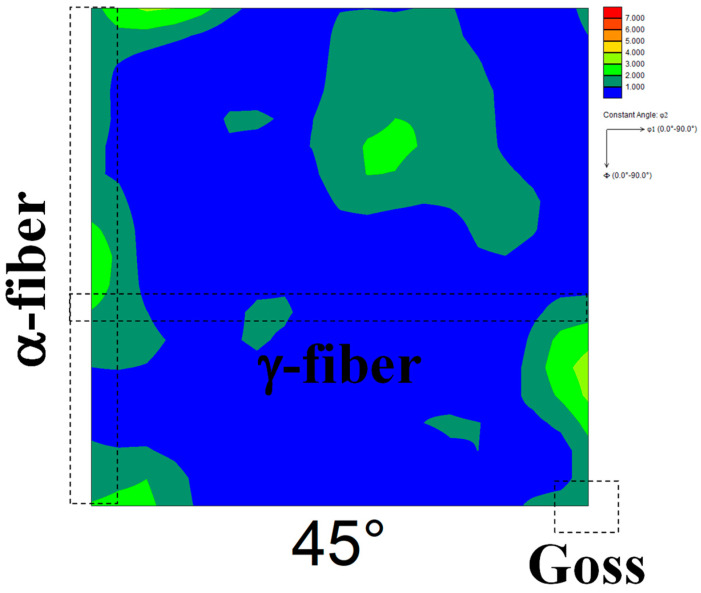
ODF map of specimen 60C annealed at 1073 K.

Figure 8 shows the ODF map of the cold-rolled specimen 60C. The cold reduction ratios in each stage in specimen 60C and the specimen used in our previous report [13,14] were the same, but the cold-rolling directions in each stage were opposite. The development of γ-fiber and α-fiber was observed irrespective of the cold-rolling direction in each stage. Figure 9 shows the ODF maps of specimen 60C annealed at 1073 K. Randomization of recrystallization texture and the emergence of Goss orientation were observed irrespective of the cold-rolling direction in each stage. These results indicate that not only the cold reduction ratio but also the cold-rolling direction in each stage hardly affects the texture evolution during cold-rolling and subsequent short-term annealing.

**Figure 10:**
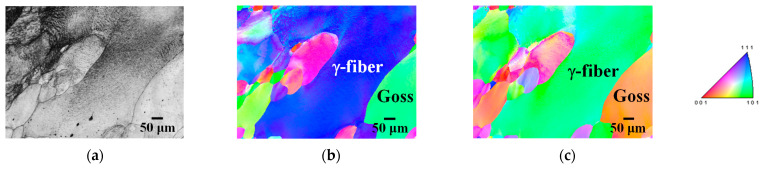
(**a**) IQ and IPF maps in the (**b**) normal and (**c**) rolling directions of specimen 60C annealed at 1123 K for 180 min.

Figure 10 shows the IPF maps of specimen 60C annealed at 1123 K for 180 min. Abnormal Goss grain growth was observed, consistent with the findings of our previous study [14]. Abnormal growth of grains with γ-fiber was also observed. In previous studies, the development of γ-fiber and α-fiber in conventional one-way cold-rolled iron and steel has been generally confirmed [30]. Following annealing, γ-fiber with high strain develops preferentially while consuming α-fiber with low strain, and eventually only γ-fiber develops [11]. In the case of specimen 60C, the cold-rolling direction in the first stage was the same as that of the as-received sheet. This means that the cold-rolling direction in specimen 60C was changed only once, whereas it was changed twice in other specimens. Thus, the effect of two-way cold-rolling on texture evolution in specimen 60C is weaker than that in other specimens. As a result, the texture of specimen 60C exhibits features of both one-way and two-way cold-rolled pure iron (γ-fiber and Goss orientation).

**Figure 11:**
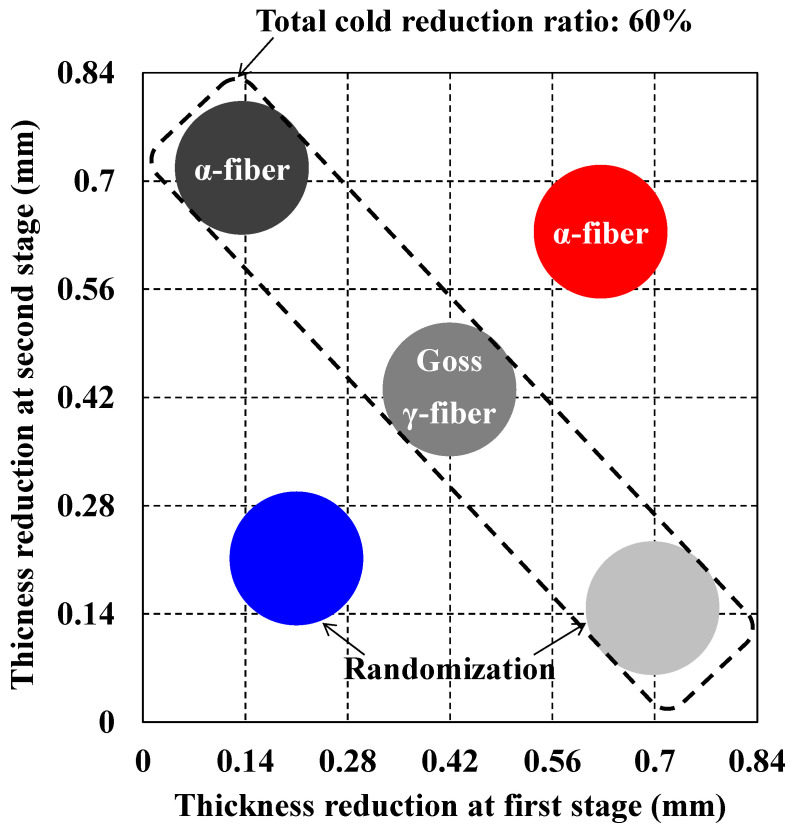
Summary of the recrystallization textures obtained from two-way cold-rolling and subsequent long-term annealing.

Figure 11 summarizes the recrystallization textures obtained via two-way cold-rolling with subsequent long-term annealing. Texture randomization was observed at low thickness reduction in the second stage, whereas the development of α-fiber was observed at high thickness reduction in the second stage. At a cold reduction ratio of 60%, abnormal grain growth was observed, but the crystal orientations of the grains varied.

### 3.4. Machine Learning Analysis

**Figure 12:**
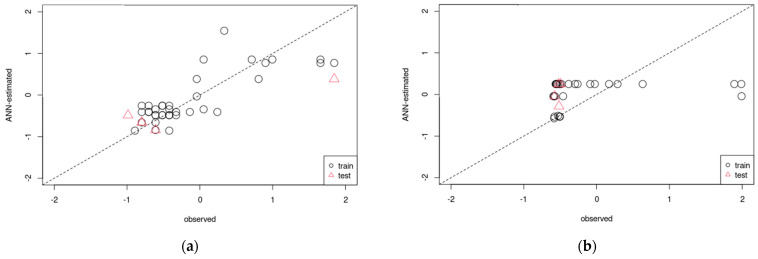
Comparison of actual and predicted values in regression models for (**a**) nucleation and (**b**) growth of Goss grains obtained by artificial neural networks (The closer the plot is to the dotted line, the higher the accuracy).

**Figure 13:**
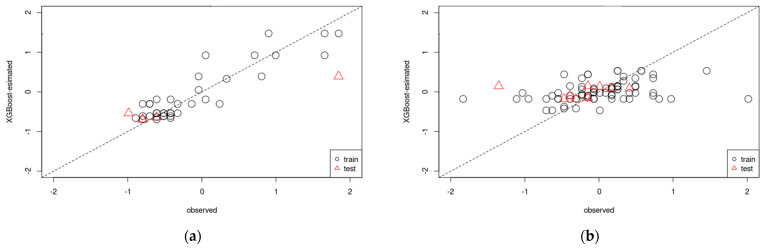
Comparison of actual and predicted values in regression models for (**a**) nucleation and (**b**) growth of Goss grains obtained by XGBoost (The closer the plot is to the dotted line, the higher the accuracy).

As mentioned above, the nucleation and abnormal growth of grains with Goss orientation are characteristic of two-way cold-rolled and annealed pure iron. Based on the experimental results, we developed regression models for Goss orientation using ANNs and XGBoost. Figure 12 and Figure 13 show the accuracy of the ANN and XGBoost regression models for Goss orientation. In both figures, the vertical and horizontal axes correspond to the estimated and experimental values, respectively. Furthermore, the regression models were constructed separately for short-term (nucleation of Goss grains) and long-term (growth of Goss grains) annealing. The coefficients of determination of nucleation (Figure 12a) and grain growth (Figure 12b) models constructed using ANN were 0.93 and 0.30, respectively. Additionally, the coefficients of determination of nucleation (Figure 13a) and grain growth (Figure 13b) models constructed using XGBoost were 0.98 and 0.053, respectively. In the case of nucleation, the accuracies of the models constructed using ANNs (Figure 12a) and XGBoost (Figure 13a) were high. However, the accuracies of the models constructed using ANNs (Figure 12b) and XGBoost (Figure 13b) for grain growth were low. This was attributed to insufficient data variability in the dataset used to construct the grain growth regression model. In particular, the dataset lacked data on the intermediate stages of abnormal Goss grain growth. Therefore, more experimental data should be accumulated to improve the accuracy of regression models for the growth of Goss grains.

**Figure 14:**
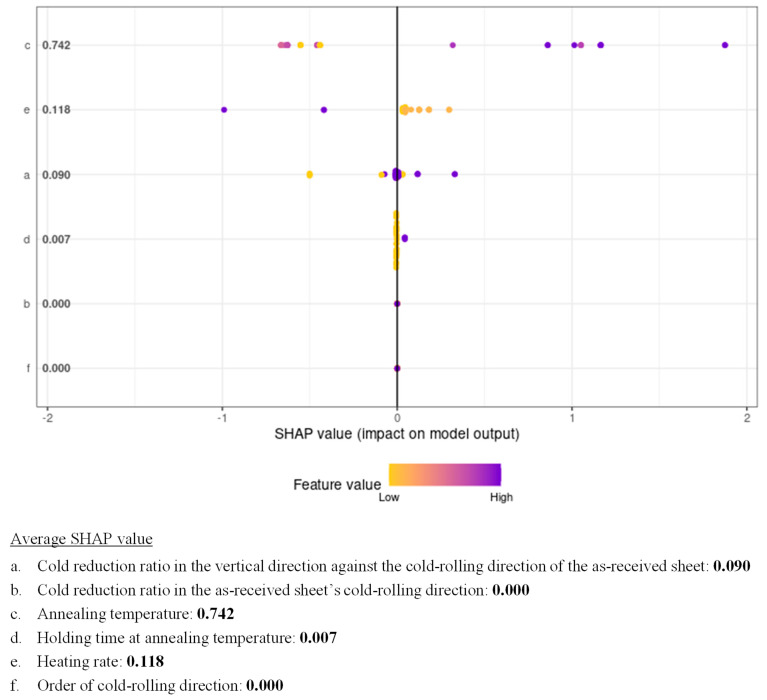
SHAP analysis results for cold-rolling and annealing conditions regarding the nucleation of Goss grains.

We quantitatively evaluated the effect of cold-rolling and annealing conditions on the degree of development of Goss orientation using sensitivity analysis of the constructed ANN model. Table 1 shows the sensitivity analysis results for cold-rolling and annealing conditions regarding the nucleation of Goss grains. The larger the value shown in Table 1, the greater the effect of the parameter on the constructed model. As shown in Table 1, the annealing temperature was the dominant factor for the development of Goss orientation. We also attempted to quantitatively evaluate the effect of cold-rolling and annealing conditions on the degree of development of Goss orientation using SHAP based on the XGBoost model (Figure 14). The SHAP value quantifies the contribution of each manufacturing process to the development of Goss orientation and is expressed as positive (right side) and negative (left side) values. Feature values correspond to the degree of Goss orientation development, and the plots are represented in blue and yellow when the feature values are high and low, respectively. As shown in Figure 14, the dominant factor for the development of Goss orientation was the annealing temperature, confirming the results of the sensitivity analysis. Recovery and recrystallization during annealing at higher temperatures are necessary for the nucleation of Goss grains [13,14]. Additionally, as shown in Figure 7 and Figure 10, grains having orientations other than Goss orientation can preferentially grow even if the annealing temperature is high. Therefore, the annealing temperature has both positive and negative effects on the prediction model. Thus, the results obtained via sensitivity analysis and SHAP are reasonable. Although two-way cold-rolling affects the nucleation of Goss grains [13,14], it is less important than the annealing temperature. These results were explained by the insufficient variety of cold-rolling conditions used in this study. Herein, the effect of cold-rolling and annealing conditions on the degree of development of Goss orientation was quantitatively evaluated using sensitivity analysis and SHAP.

## 4. Conclusions

We performed a machine learning-aided analysis of rolling and recrystallization textures in pure iron with different cold reduction ratios and cold-rolling directions. The following results were obtained:

(1) Two-way cold-rolling had a small effect on the rolling texture evolution at cold reduction ratios different from 60%.
(2) The cold reduction ratio in each stage hardly affected the texture evolution during cold-rolling and subsequent short-term annealing. In the case of long-term annealing, although abnormal grain growth occurred, the crystal orientations of the grains varied.
(3) The cold-rolling direction in each stage also hardly affected the texture evolution during cold-rolling and subsequent short-term annealing. Simultaneously, the texture of specimen 60C exhibited features of both one-way and two-way cold-rolled pure iron (γ-fibers and Goss grains).
(4) Regression models for the nucleation of Goss grains constructed using ANN and XGBoost showed high accuracy. Moreover, sensitivity analysis revealed that the annealing temperature was the dominant factor for the development of Goss orientation.

In future studies, inverse problem analysis should be performed to optimize the cold-rolling and annealing conditions for the development of each crystal orientation.

## Figures and Tables

**Table 1:** Sensitivity analysis results for cold-rolling and annealing conditions regarding the nucleation of Goss grains.

Factors	Value
Cold reduction ratio in the vertical direction against the cold-rolling direction of the as-received sheet	1.55
Cold reduction ratio in the as-received sheet’s cold-rolling direction	1.55
Order of cold-rolling direction	1.56
Heating rate	1.44
Annealing temperature	5.97
Holding time at annealing temperature	1.07

## Data Availability

The original contributions presented in the study are included in the article, further inquiries can be directed to the corresponding author.
